# Editorial Comment: Testosterone replacement therapy (TRT) and prostate cancer: An updated systematic review with a focus on previous or active localized prostate cancer

**DOI:** 10.1590/S1677-5538.IBJU.2022.01.08

**Published:** 2021-12-21

**Authors:** Valter Javaroni

**Affiliations:** 1 Hospital Federal do Andaraí Departamento de Andrologia Rio de Janeiro RJ Brasil Departamento de Andrologia, Hospital Federal do Andaraí, Rio de Janeiro, RJ, Brasil

Louis Lenfant 1, Priscilla Leon 2, Géraldine Cancel-Tassin 3, Marie Audouin 4, Frédéric Staerman 5, Morgan Rouprêt 6, et al.

Urol Oncol. 2020 Aug;38(8):661-670.

## COMMENT

Imagine you counseling a man who was successfully treated for localized prostate cancer (CaP) and now suffers from late-onset hypogonadism. What puts him more at risk: cancer or cardiovascular disease? Would there be a difference in this decision made 10 years ago?

In this nice review, authors make a journey throughout the evolution of evidence in testosterone replacement therapy (TRT) beginning with the arguments against Huggin’s dogma, passing by the true risk of CaP in hypogonadal men treated with TRT, and finally dealing with the possibility that hypogonadic patients with localized CaP could benefit from androgen therapy without compromising their chance of curing cancer. They conclude, after this long voyage: “currently, no definitive recommendation has been made for the administration of androgen therapy to patients with CaP”.

Undoubtedly, despite the lack of a definitive recommendation, there have been significant advances in this matter. Knowledge has improved in such significant topic and this paper stresses the relevance of patient’s participation on therapeutic decision. Treating middle-age men with CaP localized disease requires getting rid of old fears and developing a holistic view of men’s health that encompasses balancing the risks and benefits of adjusting testosterone (T) to normal values.

With the increasing male life expectancy worldwide and development of adequate T preparations, the prescription of T has increased tremendously. TRT in the United States tripled from 2001 to 2011, mostly in men without a clear reason (1, 2).

Hypogonadism (T deficiency) in adult men is a clinical and biochemical syndrome associated with low level of T, which may adversely affect multiple organ functions and quality of life. Hypogonadism is associated with the development of metabolic syndrome, type 2 diabetes and cardiovascular disease and can be associated with an increased mortality rate. Therefore, it must always be considered pathological when diagnosed (3).

TRT should be based on low serum T and related clinical symptoms. And, certainly, also depends on the absence of contraindications (4). Until now, most treatment guidelines recommend against the initiation of TRT in patients with a history of or known risk factors for CaP. Guidelines recommend against T replacement in patients with metastatic or locally advanced CaP and in patients at high risk for recurrent CaP (4-8).

Last guideline’s revisions show some changes on CaP as a definitive contraindication for TRT. The Endocrine Society has the strictest guidelines, advising against T in patients with an unevaluated prostate nodule, PSA >4ng/mL, or PSA >3ng/mL in high-risk patients (i.e., African Americans or first-degree relative with CaP). Only the AUA and ISSM recommend offering T on a case-by-case basis for all patients with CaP. Patients treated for localized CaP with no evidence of active disease (measurable PSA, abnormal digital rectal examination findings, evidence of bone or visceral metastases) are candidates for replacement under the Canadian Medical Association Journal, European Association of Urology (EUA), and British Society for Sexual Medicine guidelines (BSSM). However, an additional caveat for the EAU and BSSM guidelines is that there must be a low risk for recurrent CaP (Gleason score <8, pT1-2, preoperative PSA <10ng/mL). The EAU also recommends T replacement in patients treated with brachytherapy or external-beam radiation therapy for low-risk CaP (7).

However to reduce the distance between guideline’s recommendations and the daily practice, we need to understand the evolution of the evidence. The fear of prescribing T and increases the risk of prostate cancer still exists and it is based on the old concept of androgen sensitivity of tumor prostate cells. This theory, which has been updated, was primary based in a linear correlation between T and CaP that should endure regardless of the T concentration. Since the discovery by Huggins and Hodges, 79 years ago, of the hormonal dependence of CaP, the dogma still is that T stimulates CaP and that castration reduces metastatic cancers (9).

CaP not observed in eunuchs; the evidence that administration of T in men with CaP led to rapid and poor outcomes; and total androgen suppression by castration as an effective first-line treatment for advanced CaP; all these data lead to the theory that high levels of circulating androgens were a risk factor for CaP (10, 11).

With the use of luteinizing-hormone-releasing hormone (LHRH) agonist medications as a replacement for surgical castration, the idea that T was negatively linked with prostate cancer was reinforced by the notions of “T flare”, a transient rise of serum T which accompanies LHRH agonists, and was then associated with severe adverse events such as vertebral collapse, paralysis, acute urinary retention and even death (12).

It took until the 1990s, when topical T formulations were introduced to treat hypogonadism, for the testosterone-dependent model of CaP start to be seriously questioned (13).

Reports that hypogonadal men with normal PSA did not have lower cancer rates than the general male population; and the fact that approximately 1 in 7 hypogonadal men with a PSA level of 4ng/mL or less has biopsy detectable cancer - meaning that men who had CaP did not fit the model where higher T concentrations increased the risk of CaP and consequently low T level would have a protective effect - put the linear correlation in doubt. So what could explain why serum T does not appear to be related to the risk of CaP in the general population, and why the administration of T in men with metastatic CaP results in a rapid progression of the disease in castrated men, but not in eugonadal men (14)?

One possible solution for that apparent paradox was the so called “Morgentaler and Traish’s Saturation Model”: this biphasic model postulates that the CaP response to variations in T levels at castration or near castration range reaches a point of maximal prostate stimulation beyond which further increases produce little or no further effect on the prostate. The saturation model starts from the observation that CaP growth is sensitive to variation in serum T concentrations at or below the castrate range and is insensitive to T variation above this concentration. This explains the two findings that seem contradictory: while CaP is extremely sensitive to low levels of T, there is ample evidence that its growth is not influenced by androgens at higher concentrations (15).

In agreement with the Saturation Model, data collected from the Massachusetts Male Aging Study on more than 1.500 men showed no significant correlation between the risk of CaP and androgens concentration (16).

A sizable proportion of hypogonadal men has biopsy-detectable prostate cancer despite normal PSA levels. In addition, the combination of low serum T and PSA level greater than 2.0ng/mL appears to be particularly worrisome for the presence of cancer (30.2% of such men in one important study had positive biopsy findings) showing that lower T levels are associated with an increased risk of cancer (17).

Analysis of pooled worldwide data from 18 prospective studies (more than 3000 cases and 6000 controls) found no significant association between serum T concentrations and CaP risk. In fact, the evidence of those 18 studies indicates that changes to T within the physiological range have little or no effect on the prostate (both benign and malign) (18).

Meta-analysis of randomized, placebo-controlled studies investigating the association of TRT and CaP showed an association of reduced risk, albeit an insignificant one (19, 20).

A meta-analysis showed no significant association between TRT and the incidence of CaP or the need for prostate biopsy when compared with the placebo/non-intervention group (21).

In randomized controlled trials, TRT did not significantly increase the rate of CaP in T deficient older men who received active therapy compared with those who received placebo (22, 23).

In a cohort of 12.779 men who were newly diagnosed with late-onset hypogonadism use of TRT was not associated with an overall increased risk of CaP (hazard ratio=0.97; 95% confidence interval: 0.71, 1.32) compared with nonuse. Results remained consistent in secondary and sensitivity analyses, as well as in a propensity score-matched cohort analysis that further assessed the impact of residual confounding (24).

In response to widespread concerns about the treatment with exogenous T for male hypogonadism, an international conference of consensus of experts ended with unanimous approval of nine resolutions reinforcing that the evidence does not allow demonstrating an increased risk of CaP with exogenous T treatment (25).

But saturation model could explain everything? Since 1990, Grasso et al. reported that at time of CaP diagnosis, the mean serum concentration of sex hormone binding globulin (SHBG) is significantly higher in CaP patients than in men with benign prostatic enlargement or in healthy individuals (26). This finding was corroborated in another report in which high preoperative SHBG was an independent and highly accurate predictor of lymph node metastasis at radical prostatectomy (27). Because high levels of SHBG may be associated with low concentrations of bioavailable T, these studies appear to support the counterintuitive concept that low androgen levels may be related to adverse CaP outcomes (28).

Some concerns appeared after Saturation Model theory, like the risk for a possible misinterpretation with the dangerous message that continuous T administration with elevated serum levels cannot produce a risk for CaP growth, with or without CaP disease (29). And how Saturation Model alone explains the evidence that tumors arising in a low T environment appeared to be more aggressive (28, 30)?

Back to molecular level, it is known that within prostate epithelial cells, T is irreversibly converted into the primary effector androgen, 5a-dihydrotestosterone (DHT), by the enzyme 5a-reductase. DHT binds to the cytoplasmatic androgen receptor, and the DHT androgen receptor complex translocates into the cell nucleus, where it stimulates the transcription of androgen-regulated genes (31). But there are more complexity hidden here. Androgen dependence of CaP cells was set in a new light with the findings on androgen receptor alterations and expression as well as local testosterone production in CaP (32, 33).

One possible explanation of T level’s influencing CaP aggressiveness came from Chicago University’s: Intracellular androgen receptors (iAR) and membrane androgen receptors (mAR) tend to act in opposition to each other, with iAR downregulating strongly antiapoptotic proteins such as Bcl-2 and strongly proapoptotic proteins such as Fas and with mAR upregulating both Fas and, to a lesser degree, Bcl-2. The correlation between higher levels of T and less aggressive PCa could be caused by an increase in the rate of apoptosis. There is typically so many more mAR in PCa than in normal prostate epithelial cells that it has been suggested that the presence of significant numbers of mAR might be used as a diagnostic tool (34, 35).

The role of estrogen receptors α and β (ERα and ERβ) in CaP progression further increased complexity of the mechanisms (36). The expression of estrogen receptors ERα and ERβ changes in different stages of the CaP and conflicting findings on the roles of these receptors continue to emerge. Androgen-independent prostate cancer cells express both ERα and ERβ. The activation of ERβ increases the expression of β-catenin and proliferation of CaP cells. The activation of ERβ also promotes the increase of migration, invasion and anchorage-independent growth of CaP cells. Furthermore, the activation of ERα also plays a role in invasion and anchorage independent growth of CaP cells (37).

So TRT besides been safe could also have a protective effect? In a large Sweden study including 38.570 CaP cases and 192.838 age matched controls, patients who received TRT (1% of the cases and controls) had more favorable-risk CaP and lower risk of aggressive CaP in multivariate analyses (38).

The safety of TRT with respect to CaP patients was first reported by Kaufman and Grydon (39). After that, several small studies confirmed the safety of T replacement after CaP treatment either with radical surgery or radiation therapy (40-45).

Together with a growing understanding of the negative health effects and decreased quality of life in men with hypogonadism (46-48), a paradigm shift away from T as a CaP inducer occurred allowing clinicians to use TRT as potential treatment for men with difficult and symptomatic hypogonadism that had been previously treated for CaP (49).

In a systematic review and meta-analysis including 21 studies, authors did not observe higher rate of biochemical recurrence after TRT for secondary symptomatic hypogonadism in nonmetastatic CaP patients who underwent definitive local therapy with curative intent (50).

In a recent published retrospective analysis of 1303 patients who underwent robotic-assisted radical prostatectomy, 47 men with symptoms of andropause and low serum testosterone received TRT. After the follow-up period of 48 months, three (6.4%) and 157 (12.56%) patients experienced biochemical recurrence in TRT group and non TRT respectively. In the multivariate analysis, higher pre-prostate-specific antigen (PSA) (p=0.043), higher International Society of Urological Pathology score (p <0.001), seminal vesical invasion (p=0.018) and positive surgical margin (p <0.001) were predictors of recurrence. However, TRT was not (p=0.389). In addition, there was a significant change in the Sexual Health Inventory for Men (p=0.022), and serum testosterone level (p <0.001) before and 6 months after initiation of TRT. The authors concluded that TRT, in well-selected, closely followed, symptomatic men is an oncological safe and functional effective treatment in prostate cancer patients after robotic-assisted radical prostatectomy (51). The small number of men in active group and the short period of follow up limits their findings.

And even in the hormone resistant CaP the TRT has been experimentally evaluated. Although typically very effective initially, responsiveness of androgen deprivation therapy in most cases is finite. On average, patients with systemic CaP fail first line attempt within 2-3 years, progressing to castration resistant prostate cancer (CRPC), also termed androgen independent or hormone refractory CaP. Furthermore, androgen ablation is typically maintained throughout the course of therapy and is associated with significant side effects such as persistent hot flashes, osteoporosis, sexual dysfunction, metabolic/cardiac toxicities, and diminished overall quality of life (52).

The University of Chicago Medical Center in a phase I study included 15 CRPC with none to minimal metastatic disease to treatment with transdermal T. The underlying hypothesis that T may have growth inhibitory effects in patients with castrate resistant disease. There is furthermore a suggestion of anti-tumor effect based on a few patients with PSA decreases and long term disease stabilization in a subset of patients. The small sample precludes any definitive conclusion, and a larger, placebo controlled, randomized study of T in CRPC patients has been initiated to more accurately determine the effects of T on disease progression and quality of life (53). These results are similar to another report of a phase I trial of T therapy in CRPC from Memorial Sloan Kettering Cancer Center that showed that high-dose exogenous T can be administered safely to patients with castration-resistant disease (54).

But those pilot studies evoked some criticism: Treat low-risk castration-resistant CaP patients with TRT without doing anything to prevent the conversion of T to estradiol (E2) by aromatase was considered inappropriate. Ordinarily, there is no aromatase activity in normal prostate epithelial cells, but there is in CaP. Therefore, an increase in T would be expected to cause a greater increase in the local E2 level around the CaP than in the serum E2 level. Because ERα promotes CaP growth and ERβ promotes CaP death, in the short time frame of the study, the effects of E2 on the estrogen receptors may mask the effect of T on the androgen receptors. E2 can be either beneficial or harmful, depending on the initial levels of the various estrogen receptors α and β (55).

And finally, to reinforce the idea that it is not only T level that matters and, in the absence of a definitive recommendation, TRT should always be based on the presence of clinical signs and symptoms justified by hypogonadism, on the possibility of improving the quality of life while maintaining safety. Each case must be individualized, deserves a wide discussion considering that particular situation and the final decision should be shared with the patient.

In this respect, it is worth remembering about a more subtle modulation of androgen effects that is related to the repeat polymorphism (CAGn) in exon 1 of the androgen receptor gene. It influences the relation between T level and symptoms.

It is well known that transcription of androgen-dependent target genes is attenuated with increasing length of triplets. As a clinical entity, the CAG repeat polymorphism can relate to variations of androgenicity in men in various tissues and psychological traits: the longer the CAGn, the less prominent is the androgen effect when individuals with similar T concentrations are compared. Men with longer CAG repeat length and low T concentrations showed the highest risk of incident metabolic syndrome. CAG repeat length is a risk factor of incident low T concentrations and a contributing factor of testosterone-related cardiometabolic effects, including sexual dysfunctions (56, 57).
